# Multiplex detection, distribution, and genetic diversity of *Hop stunt viroid* and *Citrus exocortis viroid* infecting citrus in Taiwan

**DOI:** 10.1186/s12985-015-0247-y

**Published:** 2015-02-03

**Authors:** Chun-Yi Lin, Meng-Ling Wu, Tang-Long Shen, Hsin-Hung Yeh, Ting-Hsuan Hung

**Affiliations:** Department of Plant Pathology and Microbiology, National Taiwan University, Taipei, 10617 Taiwan; Division of Forest Protection, Taiwan Forestry Research Institute, Taipei, 10066 Taiwan; Agricultural Biotechnology Research Center, Academia Sinica, Taipei, 11529 Taiwan

**Keywords:** *Citrus exocortis viroid* (CEVd), *Hop stunt viroid* (HSVd), Multiplex RT-PCR, Multiplex real-time RT-PCR, Co-infection, Uneven distribution

## Abstract

**Background:**

Two citrus viroids, *Citrus exocortis viroid* (CEVd) and *Hop stunt viroid* (HSVd), have been reported and become potential threats to the citrus industry in Taiwan. The distributions and infection rates of two viroids have not been investigated since the two diseases were presented decades ago. The genetic diversities and evolutionary relationships of two viroids also remain unclear in the mix citrus planted region.

**Methods:**

Multiplex RT-PCR was used to detect the two viroids for the first time in seven main cultivars of citrus. Multiplex real-time RT-PCR quantified the distributions of two viroids in four citrus tissues. Sequence alignment and phylogenetic analysis were performed using the ClustalW and MEGA6 (neighbor-joining with p-distance model), respectively.

**Results:**

HSVd was found more prevalent than CEVd (32.2% vs. 30.4%). Both CEVd and HSVd were commonly found simultaneously in the different citrus cultivars (up to 55%). Results of the multiplex quantitative analysis suggested that uneven distributions of both viroids with twig bark as the most appropriate material for studies involving viroid sampling such as quarantine inspection.

Sequence alignment against Taiwanese isolates, along with analysis of secondary structure, revealed the existence of 10 and 5 major mutation sites in CEVd and HSVd, respectively. The mutation sites in CEVd were located at both ends of terminal and variability domains, whereas those in HSVd were situated in left terminal and pathogenicity domains. A phylogenetic analysis incorporating worldwide viroid isolates indicated three and two clusters for the Taiwanese isolates of CEVd and HSVd, respectively.

**Conclusions:**

Moderately high infection and co-infection rates of two viroids in certain citrus cultivars suggest that different citrus cultivars may play important roles in viroid infection and evolution. These data also demonstrate that two multiplex molecular detection methods developed in the present study provide powerful tools to understand the genetic diversities among viroid isolates and quantify viroids in citrus host. Our field survey can help clarify citrus-viroid relationships as well as develop proper prevention strategies.

## Background

Viroids are small, circular, single-stranded noncoding RNAs that only infect plants. With tiny genome sizes (246–401 nt) and simple structures, viroids are the smallest known agents that infect hosts and cause disease. Because they lack gene-encoded proteins to provide specific functions, viroids depend on host-encoded factors and enzymes for replication [1-3]. Viroid replication occurs in specific subcellular compartments and trafficking throughout the plant, leading to complete systemic infection [4-6]. Viroids are classified into two families: Pospiviroidae and Avsunviroidae. Pospiviroidae species have a central conserved region (CCR) and do not contain hammerhead ribozymes, whereas the Avsunviroidae lack a CCR but can self-cleave through hammerhead ribozymes [7].

Citrus species are natural hosts of at least seven viroids in the family Pospiviroidae: *Citrus exocortis viroid* (CEVd, genus *Pospiviroid*), *Citrus bent leaf viroid* (CBLVd, CVd-I-b, genus *Apscaviroid*), *Hop stunt viroid* (HSVd, CVd-II, genus *Hostuviroid*), *Citrus dwarfing viroid* (CDVd, CVd-III, genus *Apscaviroid*), *Citrus bark cracking viroid* (CBCVd, CVd-IV, genus *Cocadviroid*), *Citrus viroid V* (CVd-V, genus *Apscaviroid*), and *Citrus viroid VI* (CVd-VI, genus *Apscaviroid*; initially named *Citrus viroid original sample*, CVd-OS) [8-11]. CEVd induces initial bark shelling and produces subsequent sloughing symptoms on trifoliate orange (*Poncirus trifoliata* [L.] Raf.), Troyer citrange, and Rangpur lime (*Citrus* × *limonia* Osb.), all widely used as rootstocks in commercial orchards [12,13]. HSVd variants with corresponding disease being known as cachexia induces discoloration, gumming, browning of phloem tissue, wood pitting, bark cracking, and stunting symptoms in mandarin (*C. reticulata* Blanco), clementine (*C. clementina* Hort. ex Tan.), satsuma (*C. unshiu* [Macf.] Marc.), alemow (*C. macrophylla* Webster), Rangpur lime, kumquat (*Fortunella* spp.), and mandarin hybrids such as tangelo (*C. paradisi* Macf. × *C. tangerina* Hosrt. ex Tan.) [11].

In Taiwan, citrus diseases caused by CEVd (365–475 nt) and HSVd (294–303 nt) were reported decades ago [14], but no further studies have been carried out during recent years. Following up on previous findings, in this study we simultaneously detected CEVd and HSVd isolates for the first time from seven citrus cultivars. We found that HSVd is more prevalent than CEVd and that co-infection with the two viroids is common in different citrus cultivars. In addition, we determined that twig bark is the best material for sampling viroids for assays using multiplex real-time RT-PCR [15]. Furthermore, taking into account the diversity and complexity of citrus cultivars, we aligned sequences of 18 Taiwanese isolates of CEVd and HSVd with worldwide isolates and plotted their divergences against their type species to understand the diversity and evolution of the two main viroids in Taiwan, a complex mixed-citrus planted region.

## Results

### Identification of two citrus viroids by three detection methods

**Figure 1:**
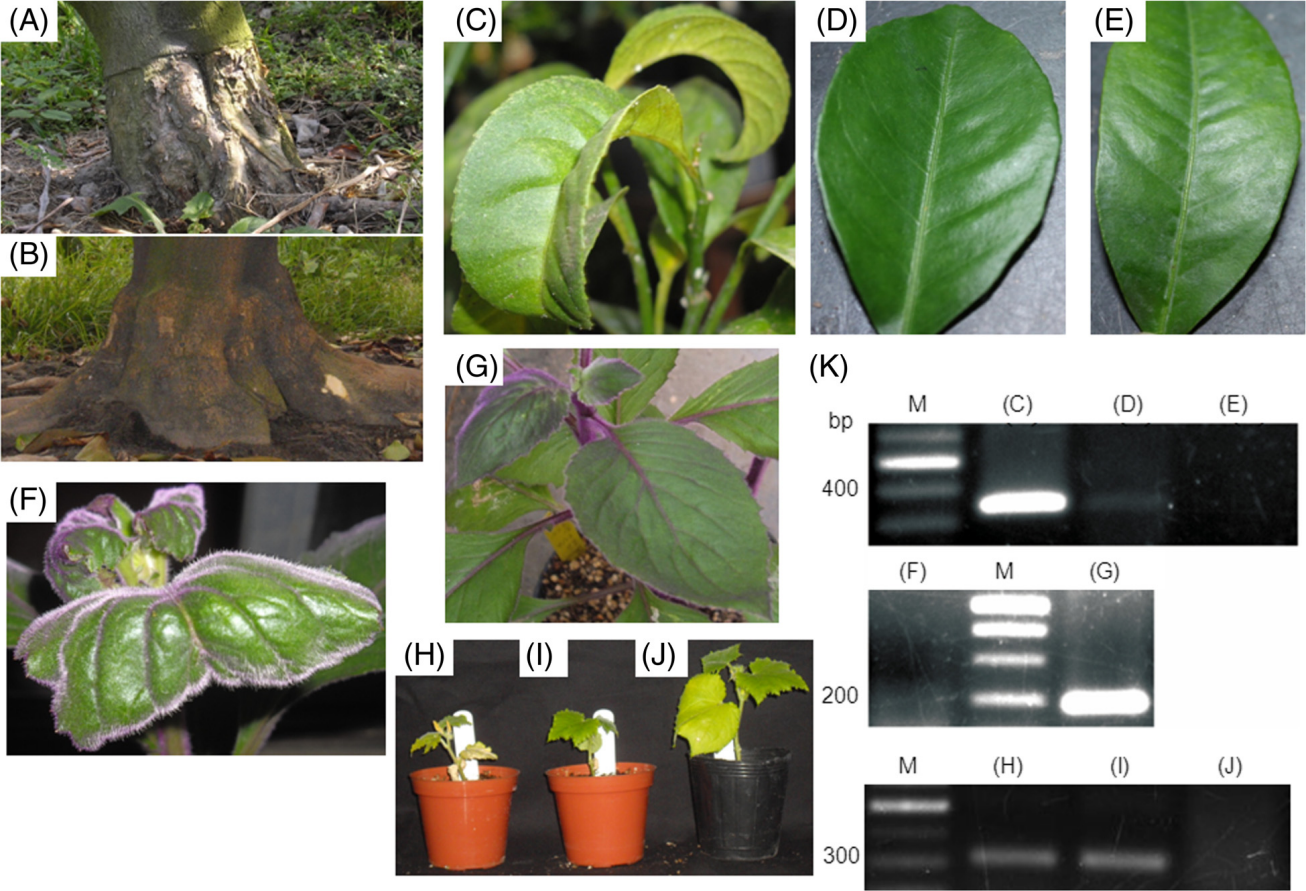
**Sensitivity of bioassay and RT-PCR methods for indicator plants infected by CEVd and HSVd.** Indicator plants as Etrog citron Arizona 861-S and *Gynura aurantiaca* were infected by CEVd; *Cucumis sativus* was infected by HSVd. Viroids resources were collected from infected field and were inoculated on indicator plants. **(A)** Viroids infected citrus tree in the field which showed typical symptoms such as exocortis and stunting compared to **(B)** Healthy citrus tree as control in the field. **(C)** Infected leaves showed symptoms such as epinasty and curling at 3 months after grafting with CEVd-infected buds. **(D)** No symptoms were shown on infected leaves at 1 month after grafting with CEVd-infected buds. **(E)** Healthy citron was a negative control. **(F)** Infected leaves showed epinasty at 3 weeks after mechanical inoculation with CEVd-infected sap. **(G)** Healthy G. aurantiaca was a negative control. **(H, I)** Infected C. sativus showed stunting after mechanical inoculation with HSVd-infected sap. **(J)** Healthy C. sativus was a negative control. **(K)** RT-PCR detection of CEVd and HSVd RNAs in infected indicator plants. M, 100-bp molecular marker; each lane represents the plants which described above.

**Figure 2:**
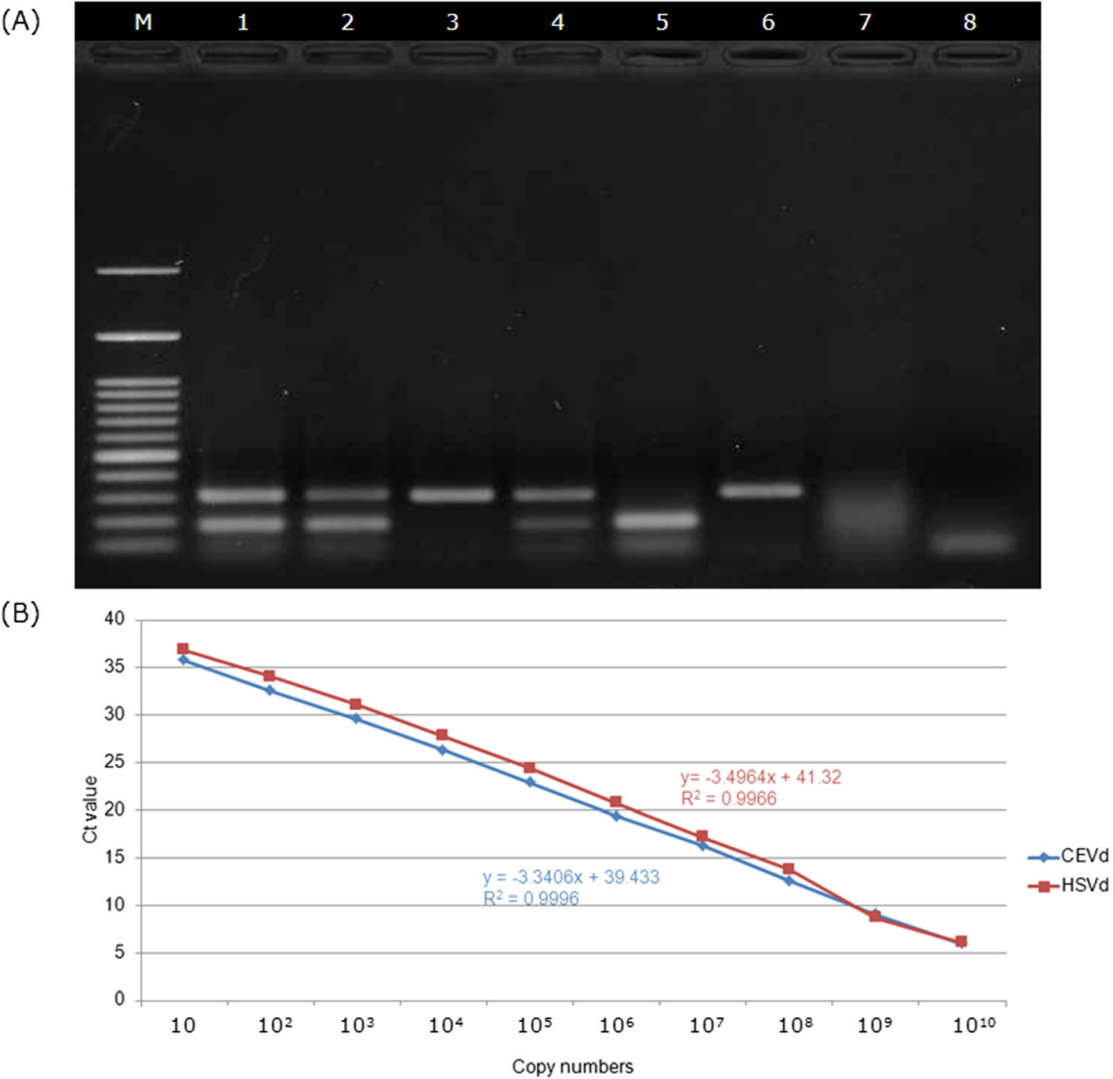
**Establishment of multiplex real-time RT-PCR and one-step multiplex RT-PCR in detecting of CEVd and HSVd. (A)** Standard curves of CEVd and HSVd for absolute quantification obtained by plotting Ct values versus actual pTOPO-CEVd and pTOPO-HSVd copy number. The Ct values for each dilution are the means of three replicates. **(B)** One-step multiplexe RT-PCR detected CEVd and HSVd transcripts alone and in combination. Samples A and B were collected from the field. Lane 1, viroids co-infection citrus sample; 2,3, unknown samples in the field; 4, Mixed 100 ng/μL RNA transcripts of plasmids of pTOPO-CEVd and pTOPO-HSVd; 5, 100 ng/μL RNA transcript of plasmid pTOPO-CEVd; 6, 100 ng/μL RNA transcript of plasmid pTOPO-HSVd; 7, healthy control; 8, water control; M, 100-bp molecular marker.

Typical symptoms of stunting and exocortis (Figure 1A) were first confirmed on citrus trees by bioassays. Three weeks after inoculation or grafting onto indicator plants, the symptoms were obvious on the main indicator plants: Etrog citron Arizona 861-S (Figure 1C) and *Gynura aurantiaca* (Figure 1F) for CEVd infection and *Cucumis sativus* for HSVd infection (Figure 1H and I). However, positive results were obtained using molecular methods at 2 weeks after inoculation, when the exterior plants still showed no symptoms (Figure 1D). According to our field observations and bioassay surveys, the two viroids existed throughout the citrus field. The development of a multiplex method that could simultaneously detect the two viroids was therefore necessary. As shown in Figure 2A, at a concentration ratio of 1:1 for the two primer sets, CEVd F194/R18 primers worked perfectly with HSVd F1/R1 primers [16] for the detection of either single or double targets. To further enhance the ability to detect early infection and to quantify viroids in citrus, we developed a real-time RT-PCR protocol for CEVd and HSVd. Samples infected by CEVd and HSVd from Yunlin County, Taiwan as well as indicator plants were analyzed and quantified by real-time RT-PCR. Ten aliquots (Figure 2B) of each viroid corresponding to 10 serial dilution concentrations were used as samples for standard curve quantification. The resulting data, such as a correlation of efficiency of 0.996-0.999 and a high amplification efficiency (92–99%), demonstrated that the standard curves were reliable for the calculation of copy numbers of unknown samples.

### Uneven distribution of viroids in different citrus tissues

**Figure 3:**
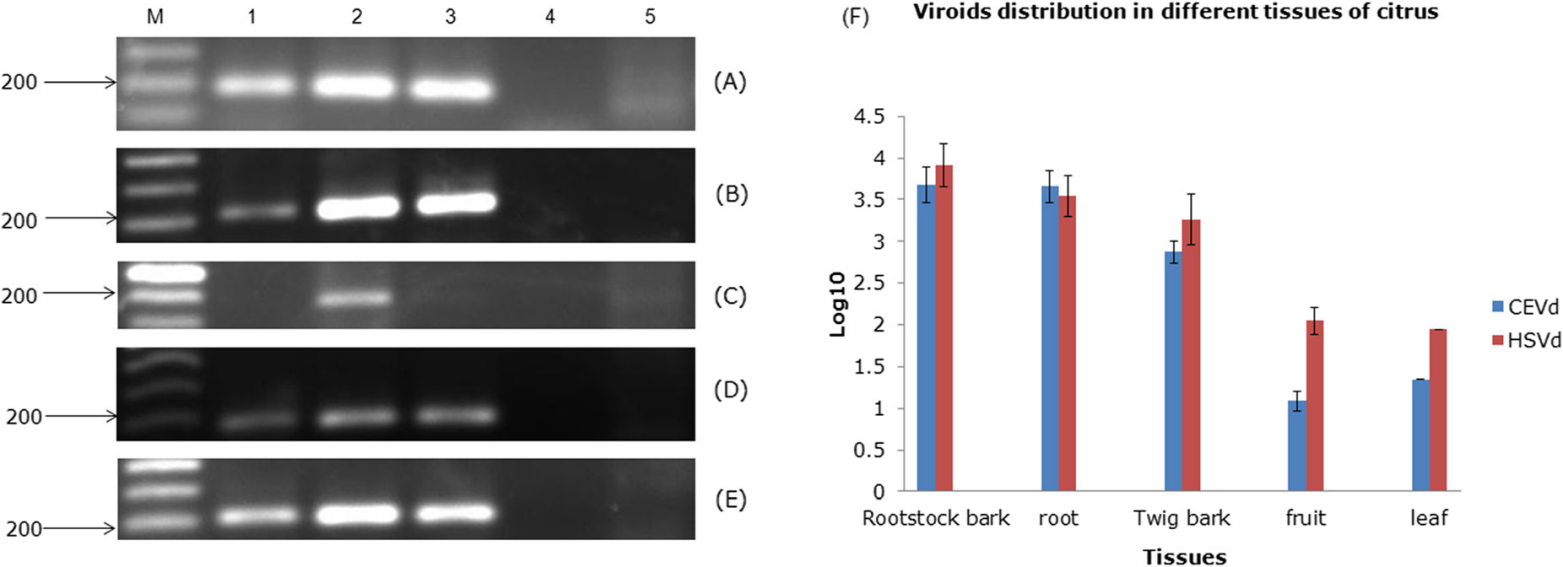
**Analysis of the viroids distribution in different citrus tissues by RT-PCR and qPCR.** For RT-PCR detection, CEVd-F194/R18 primer was used to detect CEVd from five citrus tissues. **(A)** Murcott oranges from Hsinchu; **(B)** Blood oranges from Yunlin; **(C)** Kumquat from Yilan; **(D)** Tankan from Taichung; **(E)** Lemon from Pingtung. Lane 1, exocortis symptom on rootstock; 2, root; 3, twig bark; 4, leaf; 5, healthy control; M, 100-bp molecular marker. **(F)** The quantification data of qPCR were collected among winter, 2011 to autumn, 2013 and the copy numbers of two viroids were measured by log10. The collected data of fruit were discontinuous in winter, 2011 and autumn, 2012. The collected data of leaf were only from one citrus. The bars represented the standard deviation errors.

Using multiplex RT-PCR, we analyzed the distributions of the two viroids in four citrus plant tissues. In all five citrus cultivars tested, root tissues had the highest concentrations of viroids, while leaf tissues displayed no viroid detection signals (Figure 3A-E). Quantification of the data revealed that the two viroids were similar in abundance and that both were unevenly distributed across the different citrus tissues (Figure 3F). As inferred from the log_10_-modified copy-number data, rootstocks and roots were the two regions most heavily infected by CEVd and HSVd. Leaf and fruit tissues were not included in this analysis because most of the obtained data were derived from samples in which viroids were not detected or by discontinuous sampling; consequently, most C_t_ values for these samples could not be determined by real-time RT-PCR.

### CEVd and HSVd infection rates of citrus cultivars in Taiwan

A total of 689 citrus twig bark samples were randomly collected from seven citrus cultivars in eight major citrus production counties of Taiwan. The samples were assayed by multiplex RT-PCR using the two viroid primers as described previously (Table 1). Obvious differences in infection rates were observed among regions, with the highest incidence of viroid diseases in southwestern Taiwan. The highest rates of infection were detected in lemon (89.3% infected by CEVd and 63.4% by HSVd) and blood orange (65.9% CEVd and 73.2% HSVd); the lowest were found in pummelo (*C. maxima* Merr.; 0.5% CEVd and 5.3% HSVd). On average, CEVd and HSVd infection rates were 30.4% and 32.2%, respectively. In addition, different rootstocks appeared to affect infection rates. Finally, viroid disease symptoms were frequently (up to 53.7%) due to co-infection with CEVd and HSVd, with HSVd more common than CEVd.

**Table 1:** **Multiplex RT-PCR detection of CEVd and HSVd in samples collected from different regions in Taiwan**

**Citrus cultivars**	**Locations (Number of samples)**	**Cultivars of rootstocks**	**Percentages of CEVd/HSVd infection rates (%/%)**	**Percentages of CEVd-infected/total samples (%)**	**Percentages of HSVd-infected/total samples (%)**	**Percentages of CEVd + HSVd co-infected/total samples (%)**
Tankan mandarin	Hsinchu (4)	Sour Orange	5.7/28.3	0	0	0
	Taichung (49)	Trifoliate Orange		6.1	30.6	6.1
Blood orange	Yunlin (41)	Rangpur Lime	65.9/73.2	65.9	73.2	53.7
Murcott orange	Hsinchu (61)	Sour Orange	41.5/45.8	50.8	59	39.3
	Yunlin (57)	Rangpur Lime		42.1	50.9	29.8
	Chiayi (24)	Sour Orange		16.7	0	0
Lemon	Pingtung (112)	Lemon	89.3/63.4	89.3	63.4	47.3
Wentan pummelo	Tainan (70)	Pummelo	0.5/5.3	0	0	0
	Hualien (117)			1.2	8.2	0
Kumquat	Yilan (130)	Sour Orange	9.7/9.7	9.7	9.7	9.7
Ponkan	Chiayi (24)	Sour Orange	0/0	0	0	0

### Genetic diversity and secondary structure of CEVd and HSVd isolates

Nine samples each of CEVd and HSVd from various cultivars were cloned and sequenced using CEVd dF28/dR27 and HSVd F1/R1 [16] primers. Eighteen sequences of CEVd and HSVd were deposited in GenBank under the following accession numbers: KC290927, KC290928, KJ956798-KJ956800, KJ956802-KJ956803, and KJ810543-KJ810544 for CEVd and KC290929, KJ956804-KJ956808, KJ810548, and KJ810552-KJ810553 for HSVd.

**Figure 4:**
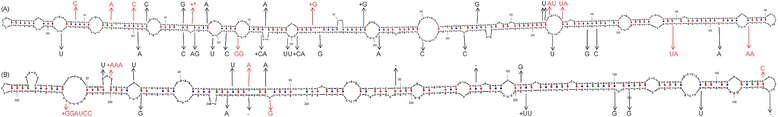
**Locations and changes of sequence variations were founded in CEVd and HSVd Taiwanese isolates.** CEVd type species **(A)**(GenBank Acc. No. KC290927) and HSVd type species **(B) **(GenBank Acc. No. KC290929) were as structure models. 17 Taiwanese CEVd isolates and 13 HSVd isolates on secondary structure were compared to each structure models. Red color nucleotides meant that the changes were found in more than three variants, whereas black color nucleotides were detected in less than two.

Alignment of the nine CEVd sequences and additional non-submitted sequences against the genome sequence of the CEVd Taiwanese type isolate (KC290927) uncovered 35 variable sites. Similarly, alignment of the nine generated HSVd sequences against the genome sequence of the HSVd type isolate (KC290929) revealed 20 mutation sites. As shown in Figure 4, Taiwanese CEVd populations contained 10 sites at which mutations (relative to the type specimen) were present in most sequences, with high overall sequence homology observed. The mutation sites existed at both ends of terminal and variability domains of the CEVd secondary structure. In addition, Taiwanese HSVd populations possessed five sites frequently undergoing point mutations or sequence insertions/deletions (indels) and exhibited high overall sequence homology. The mutation and indel sites were present on the left terminal and pathogenicity domains of the HSVd secondary structure.

### Phylogenetic analysis

**Figure 5:**
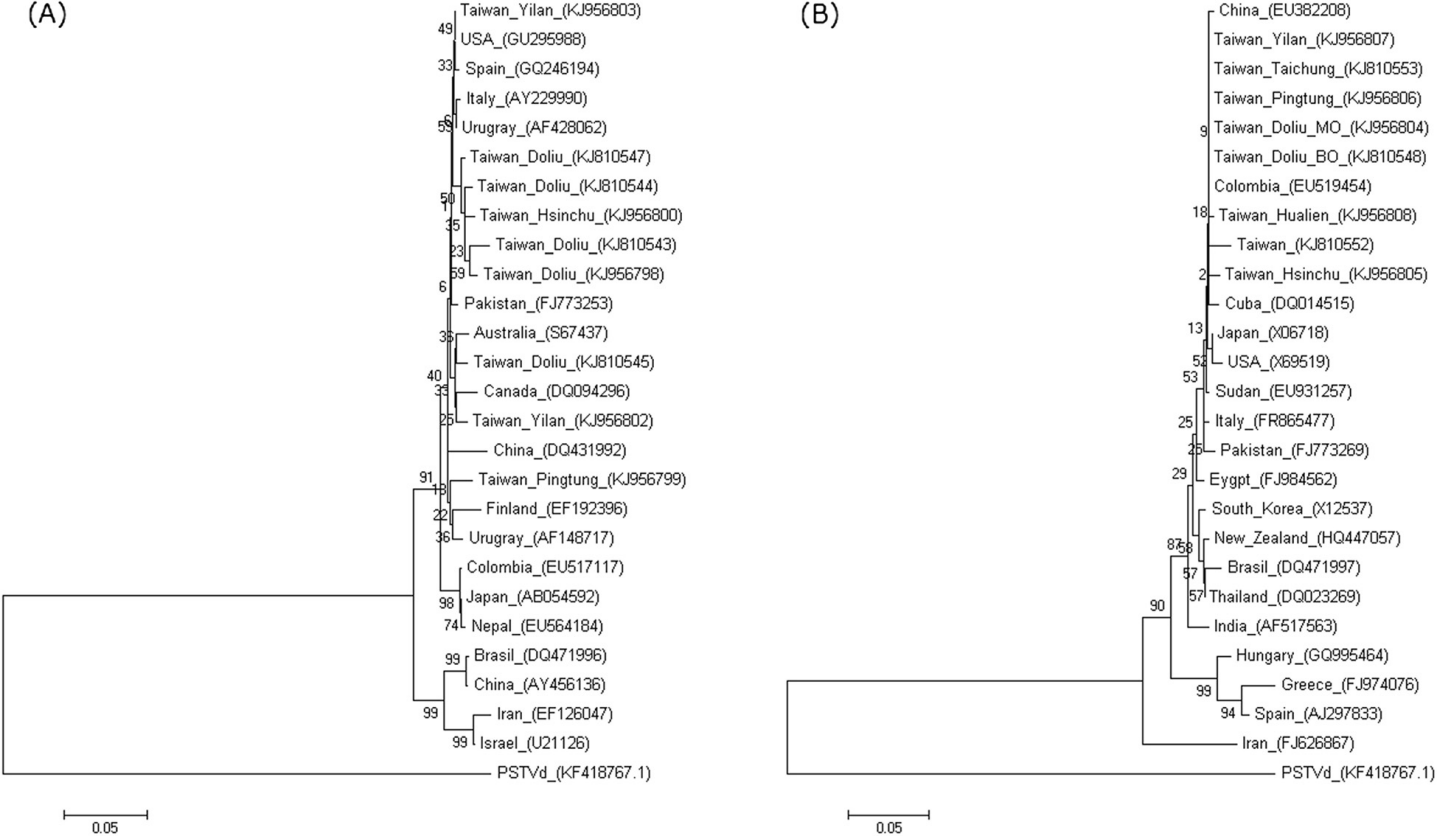
**Phylogenetic relationships of sequence variants of CEVd and HSVd.** The CEVd **(A)** and HSVd **(B)** sequence variants were obtained from Taiwan and the representative isolates of the world. Trees were constructed by MEGA 6.0 using the neighbor-joining method with 1000 bootstrap replications. *Potato spindle tuber viroid* (PSTVd) from the same family Pospiviroidae (KF418767.1) was as outgroup. Bars indicated numbers of nucleotide substitutions per site.

Phylogenetic analyses were conducted on two data sets: nine CEVd sequences generated in this study combined with 17 other worldwide CEVd sequences and eight generated HSVd sequences combined with 18 HSVd sequences from worldwide isolates. As shown in Figure 5, the nine CEVd Taiwanese isolates were distributed among three clusters and the eight HSVd Taiwanese isolates fell into two different groups.

## Discussion

### Current status of citrus diseases caused by infection with the two main viroids in Taiwan

Since the first reports of citrus diseases caused by CEVd or HSVd several decades ago, no studies have been performed on viroid diseases in Taiwan. The initial aim of our study was to fill in knowledge gaps and better understand distributions of these viroids in the citrus industry. Our field observations suggest that incorrect diagnoses are frequently made, as most viroid-infected citrus plants had latent infections without obvious external symptoms, except in sensitive rootstock 5 years old or younger. Based on our preliminary test, we tested four RNA extract methods as TRIzol buffer isolation, total nucleic acid extraction, TENS buffer extraction and purification by commercial kit (MACHEREY-NAGEL, GmbH & Co. KG, Düren, Germany). The TRIzol buffer isolation method is the best extraction method to purify viroids RNA (data not shown). When mass quantities of quarantined samples need to be assayed, multiplex RT-PCR and real-time RT-PCR are more efficient and specific for viroid detection than are field observations. In addition, the location of pathogens in infected hosts is the main issue when quarantine is required. Certain pathogens showed an uneven distribution in infected hosts; for example, *Candidatus* Liberibacter, which causes Huanglongbing in citrus, is limited to the phloem [17], and two beet viruses are distributed in different tissues of sugarbeet seedlings [18]. Another previous study also indicated that the concentrations of several viroids as CEVd, HSVd or ASSVd may be varying and uneven when infect different hosts such as citrus, pear and hop [19]. We estimated viroid distribution by real-time RT-PCR. This analysis revealed that both CEVd and HSVd were present in the lower parts of citrus plants, including roots and rootstocks where exocortis symptoms were found. Although roots and rootstock bark are the most abundant viroid-containing tissues, collection of these materials is time-consuming and difficult and the wounds may be contaminated with other pathogens. Taking into account sampling efficiency and convenience, we recommend using twig bark for viroid detection. In contrast, neither single-step RT-PCR nor qPCR could be successfully used to detect the two viroids present in high concentrations and copy numbers in leaf tissue. We found that viroids were more abundant in young tomato and citrus leaves than in mature ones (data not shown). Previous research has shown that members of different viroid genera, such as *Potato spindle tuber viroid* and *Peach latent mosaic viroid*, have opposite reactions when infecting shoot apical organs [20,21]. The cited studies demonstrate that mechanisms such as RNA silencing in leaves may operate to prevent viroid replication or transmission; nevertheless, additional evidence is required to support this hypothesis. Previous studies using multiplex RT-PCR for the field detection of citrus-infecting viroids have exploited a mixture of several primer sets in different concentrations [22,23]. Our method, which was focused on two main viroids, was developed to use a less complicated set of conditions. Our preliminary investigation yielded several discoveries. First, different cultivars and rootstocks may play an important role in viroid-host interactions and can increase citrus resistance or sensitivity, as they exhibited a range of disease scales upon viroid infection. For example, citrus plants grafted onto Rangpur lime rootstock displayed severe exocortis and stunting in Yunlin County, whereas pummelo in Hualien County showed no symptoms. Second, because mechanical inoculation is crucial to viroid transmission, viroid disease severity varied significantly according to crop management and field sanitation practices. In our experience, late-season field management was associated with the greatest severity of viroid disease. Third, most infected citrus trees showed latent infections and no symptoms unless the farmer used sensitive cultivars as rootstock. Another interesting finding was that simultaneous infection of individual citrus hosts with various viruses and viroids is common and severe in Taiwan. This situation is due to mixed crop plantings, where unknown interactions between each virus and viroid complicate detection of single diseases. Finally, slight differences in viroid sequences uncovered among citrus cultivars indicate that viroids use nucleotide and conformational alterations to adapt to different hosts. We found evidence of rapid mutation in viroids, with 2–5% divergence uncovered even within one host. Preliminary findings from our field survey were that nearly 35% of plants were infected with CEVd and HSVd viroids. The potential threat posed by viroid infection in citrus has been greatly underestimated. Regular field surveys are therefore necessary.

### Genetic variation and diversity in Taiwanese CEVd and HSVd

Although CEVd and HSVd have existed in Taiwan for decades, no molecular analyses have been performed. In this study, we randomly selected nine isolates each of CEVd and HSVd from different citrus cultivars in various areas. One isolate each of the two viroids from Yunlin County (KC290927 and KC290929) were collected as Taiwanese type isolates for further analysis. According to the alignment analysis (data not shown), CEVd isolates from different cultivars had 93–99.4% identity, whereas HSVd isolates from various cultivars were 91.2-100% identical. Among analyzed CEVd isolates, the greatest sequence divergence was observed between Murcott orange and kumquat cultivars. Surprisingly, even when CEVd isolates were collected from an area in which both Murcott and blood oranges were growing, a high level of divergence was found between isolates. In contrast, HSVd isolates were most diverged between Tankan mandarin and Murcott orange. Interestingly, identities among HSVd isolates were lower on average than those among CEVd isolates, suggesting that the two viroids may experience different mutation rates. Previous studies have shown that viroids have higher mutation rates than other microbes [24]. Even on a single citrus host, viroid populations are preserved as a genetic pool. High mutation rates may thus be triggered by different hosts or geographic factors, leading to more difficult, unreliable, and unstable detection conditions than for other pathogens. While the results of our sequence analysis might support this view, further investigations must be carried out, such as on viroid evolution in single hosts and viroid mutation among different citrus cultivars.

With respect to plotting of predicted secondary structures, we aligned all generated or downloaded CEVd and HSVd sequences against their Taiwanese type isolates. We identified 35 mutation sites, of which 10 were present more than three times among these isolates. The 10 major mutation sites were visualized on the plotted secondary structures, where they were distributed in both end terminals and variability domains as described in a previous study [25]. Interestingly, most of the CEVd sequence variants exhibited five nucleotide substitutions or indels relative to the type sequence (KC290927): U19C, U27A, A32C, −74G, and G130A. In contrast, 20 mutation sites or substitutions were present among the HSVd sequences, but only five sites were repeated more than three times among them. Five sites were located on left terminal and pathogenicity domains of the secondary structure. Almost all of the HSVd sequence variants featured three unique nucleotide substitutions or indels compared with the type sequence (KC290929), namely G26AA, G53-, and U246G. The presence of these mutations, especially those in the pathogenicity domain, is interesting, as their occurrence may be responsible for the fact that HSVd Taiwanese isolates cause scarcely any symptoms in certain hosts. Additional experiments are required to confirm which mutated position is able to affect symptoms of HSVd infection. Finally, the insertion -288GGATCC was observed in four of the aligned HSVd isolates; this additional sequence was derived from the *Bam*HI restriction enzyme cleavage site and is an experimental artifact of the cloning procedure.

In the phylogenetic analysis, nine CEVd isolates and eight HSVd isolates were analyzed along with selected worldwide isolates or variants. The CEVd Taiwanese isolates were distributed among three clusters, whereas the HSVd Taiwanese isolates fell into two separate clusters. Of the three CEVd clusters, one was most similar to Canadian (DQ094296) and Australian (S67437) strains, one was most closely related to a USA strain (GU295988) and the third was similar to a Uruguayan strain (AF148717). Interestingly, two isolates collected from kumquat in Yilan County were placed in separate clusters, suggesting that CEVd isolates may be quite different even when present in the same host. In the phylogenetic tree of HSVd sequences, one cluster was identical to a Chinese strain (EU382208) and the other to a Colombian strain (EU519454). A previous study found that AGVd isolates from India were more closely related to Chinese strains, suggesting that geographic proximity is an important factor shaping genetic similarity [26] and another studies also addressed the species-dependency of the population structure of six viroids while infecting grapevines in China and Japan [27]. In our study, however, no relationship between geographical region and genetic variation was apparent. To clarify the evolutionary origin of these viroids, additional sequences of worldwide isolates should be aligned and analyzed.

## Conclusions

We used three methods to detect two major viroids infecting citrus plants in Taiwan. Multiplex real-time RT-PCR revealed that the two viroids had similarly uneven distributions in different citrus tissues and suggested that twig bark may be the best material for sampling. Our multiplex RT-PCR-based field survey also gave preliminary estimates of CEVd and HSVd infection of nearly 30-35% in all citrus orchards in Taiwan. Sequence alignment against Taiwanese type isolates and secondary structure analysis revealed that 10 mutation sites were distributed on both ends of CEVd terminal and variability domains while five mutation sites existed in HSVd left terminal and pathogenicity domains. Finally, phylogenetic analyses incorporating worldwide viroid isolates indicated that Taiwanese isolates of CEVd are separated into three clusters and HSVd isolates form two clusters. Citrus diseases caused by viroids are a rising threat in the citrus industry in Taiwan. The results of this study should provide a solid basis for exploration of the two citrus viroids and for understanding the relationship between viroids and citrus plants. The fact that nearly 35% of citrus plants in Taiwan are infected by a combination of CEVd and HSVd suggests that viroid diseases will become an increasingly important and urgent global issue. Further studies must be performed in the near future and should focus on the ecology and epidemiology of viroids.

## Materials and methods

### Plant materials and viroid sources

**Figure 6:**
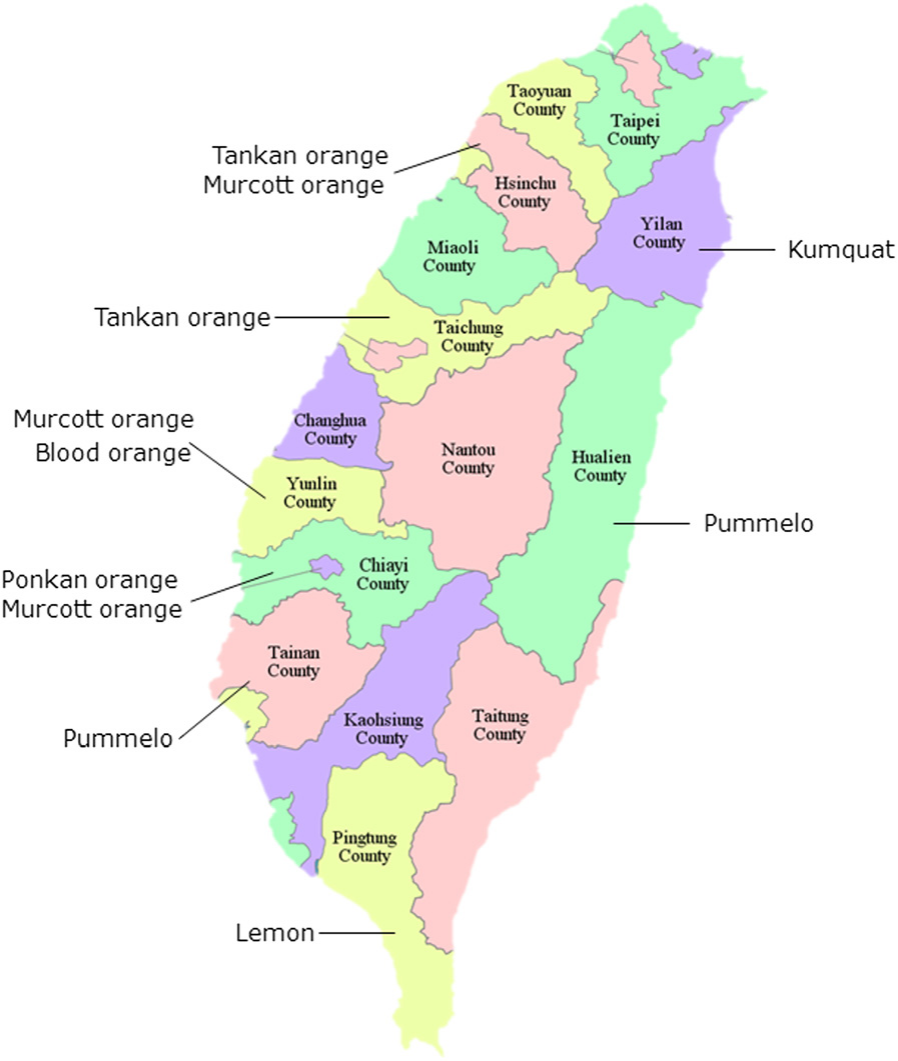
**The map showing different citrus cultivars regions used for field survey in Taiwan.** Samples were collected from Hsinchu, Taichung, Yunlin, Chiayi, Tainan, Pingtung, Yilan and Hualien counties, which contains seven citrus cultivars of citrus production.

Seven citrus cultivars, Tankan mandarin (*Citrus tankan* Hayata), Ponkan mandarin (*Citrus reticulata* Blanco), blood orange (*Citrus sinensis* var), Wentan pummelo (*C. grandis* f. buntan Hay.), Murcott orange (Climentine × *Citrus sinensis* Osbeck), lemon (Citrus × limon (L.) Burm. f.) and kumquat (*Fortunella margarita* Lour. Swingle), were randomly collected 689 trees from orchards in eight counties (Hsinchu, Taichung, Yunlin, Chiayi, Tainan, Yilan, Pingtung and Hualien) in Taiwan for field survey (Figure 6). Healthy citrus plants were obtained from pathogen-free seedlings, and viroid-infected citrus from the field were grafted to Rangpur lime (*Citrus limonia* Osbeck) for viroid preservation. The experimental plants and the indicator plants Etrog citron Arizona 861-S, *G. aurantiaca* for CEVd and *C. sativus* for HSVd were maintained in an insect-free plant greenhouse.

### Bioassay

The three repeat indicator plants Etrog citron Arizona 861-S and *G. aurantiaca* for CEVd were planted for stem cutting and grafting, respectively; the indicator plant *C. sativus* for HSVd was grown by germination from seeds (KNOWN-YOU Seed Co., Taiwan). For mechanical inoculation, infected citrus leaves from the field were ground in a 0.5 M K-P buffer (0.4 M K2HPO4, 0.08 M KH2PO4, 0.01 M EDTA-2Na, 1 mM NA2SO4, pH 7.5) as inoculum sap with carborundum was spotted onto three mature leaves of each plant by use of cotton swabs. For graft inoculation, plants with at least two infected buds, shoots and petioles were grafted with one healthy 861-S plant. Inoculated and healthy plants were grown in a greenhouse with 16 h light (28°C) and 8 h dark (24°C) cycles. Plants were regularly watered with commercial plant nutrients and checked for symptoms.

### TRIzol RNA isolation method

This method was described by [28] with some modification. Citrus tissue (100 mg) was homogenized using pestle and liquid nitrogen in 1 mL TRIzol reagent (0.8 M guanidine thiocyanate; 0.4 M ammonium thiocyanate; 0.1 M sodium acetate, at pH 5.0; 5% glycerol; and 38% phenol in a saturated buffer). After centrifugation (13,500 × g) for 10 min, the supernatant was added a 1/5 volume of chloroform, shaken vigorously for 15 s and placed at room temperature for 3 min. The supernatant was collected and added a 1/2 volume of isopropanol and 1/2 volume of 0.8 M sodium citrate/1.2 M NaCl and then placed at room temperature for 10 min. The RNA pellet was collected by high-speed centrifugation (17,000 × g) at 4°C for 15 min and then washed with 70% ethanol and resuspended in 50 μL DEPC-water.

### Sequences of primer pairs for sequencing and multiplex detection methods

All primer sets are listed in Table 2. Multiple sequence alignments of 214 sequences of CEVd (two sequences from Taiwan were included and uploaded to GenBank with accession numbers KC290927 and KC290928) involved the use of MUSCLE [29] as implemented in MEGA 6 [30]. The primer pair CEVd F194/R18 was used for detection of CEVd by multiplex RT-PCR and the pair CEVd dF28/dR27 were used for amplification of full-length CEVd by sequencing or real-time RT-PCR. The primer pair HSVd F1/R1 [16] was used for both detection and full-length sequence amplification of HSVd by sequencing, multiplex detection methods.

**Table 2:** **Details of the primers used for the molecular detections and quantifications of CEVd and HSVd**

**Primer pairs for multiplex RT-PCR**					
**Primer name**	**Sequence 5′-3′**	**Product size (nt)**	**Position**	**Description**	**References**
CEVd-dF28	GCTCVCCYGACCCYGCR^a^	371	28-44	Full-length cDNA for sequencing and qPCR	This work
CEVd-dR27	HCCACAGGRACCTCAAG^a^		11-27		
CEVd-F194	TTTCGCTGCTGGCTCCACA	196	194-212	RT-PCR	
CEVd-R18	ACCTCAAGAAAGATCCCGA		371-18		
HSVd-F1	GGGGCAACTCTTCTCAGAATCC	302	81-102	RT-PCR	[8]
HSVd-R1	GGGGCTCCTTTCTCAGGTAAGTC		58-80		
**Primers/Probes for multiplex real-time RT-PCR**					
**Primer name**	**Sequence 5**′-3′	**Product size (nt)**	**Position**	**Description**	**References**
CEVd-RTR_F	GTCGCCGCGGATCACT	64	142-159	Real-time PCR	This work
CEVd-RTR_R	CCAGCAGCGAAAGGAAGGA		187-205		
HSVd-RTR_F	GGAATTCTCGAGTTGCCGCA	127	5-24		
HSVd-RTR_R	CCGCGGCCCTCTCT		118-131		
**Probe name**	**Sequence** **5′-3′**		**Position**	**5′-Labeled**	
CEVd-RTR_P	CCAGCGGAGAAACAG		163-177	FAM	
HSVd-RTR_P	CAACTCTTCTCAGAATCC		85-102	IC	

^a^R = AG, Y = CT, H = ACT, V = ACG.

### Amplification and sequencing of viroid cDNA

Complementary DNAs were synthesized by M-MLV reverse transcriptase enzyme (Invitrogen, Carlsbad, CA, USA) using primers as described previously according to the manufacturer’s description. The fragment of viroid cDNA was then amplified by polymerase chain reaction (PCR) using Taq polymerase (Gibco BRL) in each primer pairs as described previously. The error rate of Taq polymerase is 2.2 × 10^−5^. Amplification products were analyzed on 1.6% agarose gel electrophoresis. The DNA fragments were cloned into pCR2.1 TOPO (Invitrogen, Carlsbad, CA, USA) and 5–10 clones of each fragment were sequenced with the use of the ABI PRISM automated DNA sequencer (Applied Biosystems). The nucleotide sequences were analyzed and blasted against each other by the Clustal W method using MEGA 6 software [30].

### Multiplex detection methods

#### Multiplex RT-PCR

Multiplex RT-PCR was performed in a single tube with 25 μL of the reaction mixture containing 45 mM Tris–HCl (pH 8.3), 80 mM KCl, 4 mM MgCl2, 0.2 mM dNTP, 5 mM 1, 4-dithiothreitol (DTT), 5 pmol of CEVd and HSVd primer pairs as mentioned above, 50 units of reverse transcriptase (Superscript II RNase H Reverse Transcriptase), 25 units of Taq DNA polymerase (Gibco BRL), and a 300 ng template of the nucleic acid. The thermal cycle conditions were one cycle at 50°C for 35 min; one cycle at 94°C for 2 min; 40 cycles at 94°C for 30 s, 58°C for 30 s, 72°C for 60 s; and then 72°C extension for 8 min. Reactions were carried out in a DNA Thermal Cycler 2720 (Applied Biosystems).

### Multiplex real-time RT-PCR

#### Primers and probes

All primers and probes of the two viroids are listed in Table 2. Conserved regions of probes were identified and sent to Applied Biosystems Co. (Foster City, USA) for primer and probe designations. The 5′ terminal reporter dyes were FAM (6-carboxyfluorescein) for CEVd probe; VIC for HSVd probe, and the 3′ quencher dyes were NFQ (non-fluorescent quencher) and MGB (minor groove binder).

### Standard curve and TaqMan assay setup

The standard curves for absolute quantification of CEVd and HSVd were established as described by the manufacturer (Applied Biosystems). The final concentration ranges of standard curves of CEVd and HSVd were measured from 1010 to 10 copies by serial dilution. The full-length of CEVd and HSVd were amplified using the CEVd dF28/dR27 and HSVd F1/R1 (Table 2) primer pairs and cloned into the pcr2.1-TOPO vector (Invitrogen, Life technologies, Carlsbad, CA, USA), respectively. The measurement of plasmid concentration was recorded as the absorbance at 260 nm and the copy number was calculated using the plasmid’s molecular weight and Avogadro’s constant. Synthesis of first strand cDNA for real-time PCR was followed by methods described in the above mentioned. The TaqMan PCR reactions (total volume 20 μL) were set up in 48-well reaction plates using PCR Master Mix reagent kits (Applied Biosystems). The real-time PCR conditions followed Applied Biosystems’ instruction with slight modification: 9 μL (100 ng) of cDNA, 10 μL 2X TaqMan® Gene Expression Master Mix, 1 μL 20X assay mix for each CEVd and HSVd (900 nM forward primer, 900 nM reverse primer, 250 nM TaqMan® MGB probes), and ROX passive reference dye (concentration undisclosed by manufacturer). The assays were carried out on an ABI StepOne Real-Time PCR System (Applied Biosystems) at cycling conditions (50°C /2 min, 95°C /10 min and 40 cycles of 95°C /15 min, 60°C /1 min).

### Secondary structure prediction and phylogenetic analysis

All the sequences of CEVd or HSVd were aligned with each type isoaltes (GenBank Acc. No. KC290927 and KC290929). Possible secondary structures of two viroids were predicted using the software tool as Mfold (http://mfold.rna.albany.edu/?q=mfold) and sequence variants in Taiwan were plotted against each secondary structure. All sequences were aligned with the others of randomly selected CEVd or HSVd sequences which submitted in the GenBank database using the ClustalW method and phylogenetic analysis were performed using neighbor-joining with p-distance model of MEGA6 software [30].
